# A Delphi Process to Identify Relevant Outcomes That May Be Associated With a Predictive Analytic Tool to Detect Hemodynamic Deterioration in the Intensive Care Unit

**DOI:** 10.7759/cureus.50169

**Published:** 2023-12-08

**Authors:** Andre L Holder, Ashish K Khanna, Michael J Scott, Sarah C Rossetti, Joseph B Rinehart, Dustin D Linn, Jochen Weichert, R. Philip Dellinger

**Affiliations:** 1 Critical Care Medicine, Emory University School of Medicine, Atlanta, USA; 2 Anesthesiology, Wake Forest School of Medicine, Winston-Salem, USA; 3 Anesthesiology, University of Pennsylvania, Philadelphia, USA; 4 Biomedical Informatics and Nursing, Columbia University Medical Center, New York, USA; 5 Anesthesiology, University of California, Irvine, Irvine, USA; 6 Hospital Patient Monitoring, Philips Research North America, Cambridge, USA; 7 Clinical Development, Philips Research Netherlands BV, Eindhoven, NLD; 8 Critical Care Medicine, Cooper University Hospital, Camden, USA

**Keywords:** delphi method, decision support system, hemodynamic, artificial intelligence and machine learning, adult intensive care

## Abstract

Background

The critical care literature has seen an increase in the development and validation of tools using artificial intelligence for early detection of patient events or disease onset in the intensive care unit (ICU). The hemodynamic stability index (HSI) was found to have an AUC of 0.82 in predicting the need for hemodynamic intervention in the ICU. Future studies using this tool may benefit from targeting those outcomes that are more relevant to clinicians and most achievable.

Methods

A three-round Delphi study was conducted with a panel of 10 critical care physicians and three nurses in the United States to identify outcomes that may be most relevant and achievable with the HSI when evaluated for use in the ICU. To achieve criteria for relevance, at least 65% of panelists had to rate an outcome as a 4 or 5 on a 5-point scale.

Results

Nineteen of 24 outcomes that may be associated with the HSI achieved consensus for relevance. The Kemeny-Young approach was used to develop a matrix depicting the distribution of outcomes considering both relevance and achievability. “Reduces time spent in hemodynamic instability” and “reduces times to recognition of hemodynamic instability” were the highest-ranking outcomes considering both relevance and achievability.

Conclusion

This Delphi study was a feasible method to identify relevant outcomes that may be associated with an appropriate predictive analytic tool in the ICU. These findings can provide insight to researchers looking to study such tools to impact outcomes relevant to critical care practitioners. Future studies should test these tools in the ICU that target the most clinically relevant and achievable outcomes, such as time spent hemodynamically unstable or time until actionable nursing assessment or treatment.

## Introduction

Patients in the intensive care unit (ICU) often suffer from organ failure or are at risk of organ failure [1]. Hemodynamic instability is a syndrome in which blood flow and/or perfusion to vital organs is inadequate to meet cellular metabolic demands. Failure to rescue is common in post-operative patients [2], and early recognition may be a key to prevention. Early intervention in patients with hemodynamic instability may be associated with improved outcomes. In patients with septic shock, early initiation of vasopressors has been associated with a faster time to blood pressure stability and lower mortality [3,4]. Interventions to reverse hemodynamic instability commonly include vasopressors, inotropes, bolus fluid administration, and blood transfusions.

Clinical decision support systems have the potential to monitor patients and identify those at risk of deterioration continuously; however, tools to predict deterioration are not always associated with improved outcomes [5]. The Hemodynamic Stability Index (HSI) is a machine learning model developed using 33 variables routinely obtained in the ICU to predict the need for intervention due to hemodynamic instability [6]. This model was found to have an AUC of 0.82 in predicting the need for hemodynamic intervention one hour in advance and have an AUC of 0.88 in predicting the need for vasopressor administration alone [6] and outperforms single indicators for deterioration [7].

Artificial intelligence (AI) tools may have several different applications in the ICU, including early disease identification, evolution prediction, phenotyping, and guidance of clinical decisions [8]. The HSI as a variable continuously updated at the bedside could be utilized for any of these purposes, potentially leading to further diagnostic workup, intervention or preparation for intervention, or patient triage. However, the value the end user might see in this tool is unclear. Determining the potential value of AI tools to clinicians is an important initial step in evidence generation so that these tools can be studied meaningfully. The applications of these tools should aid clinicians in improving patient outcomes, enhancing workflow, and improving the appropriateness of escalation and de-escalation of care to optimize resources. To remove potential barriers to adoption, it is first important to understand which outcomes associated with the tool may be most relevant to clinicians. Targeting the most relevant outcomes in generating evidence can help ensure the tool has clinical application. Studies can then be designed to target the most relevant outcomes to ensure clinical relevance to facilitate adoption.

The Delphi method is a consensus method that aims to achieve general agreement of opinion on a topic [9]. This technique has recently been utilized to develop a core outcome set for trials in anesthesia and perioperative medicine [10]. Here, we have used a three-round Delphi method to identify outcomes that if associated with the HSI would be most relevant to clinicians. We sought to determine the most relevant clinical, operational, and population-based outcomes. As a last step, we also sought to determine the outcomes clinicians felt most achievable in clinical studies.

## Materials and methods

We conducted a three-round Delphi study in which panelists ranked the relevance of outcomes to clinical practice for a tool that detects early hemodynamic instability. Panelists were critical care experts with or without additional expertise in predictive analytics and machine learning. The panelists, ten physicians, and three nurses were identified and recruited by the Philips Hospital Patient Monitoring Medical Science Liaisons. The survey was distributed via Microsoft Forms (Albuquerque, NM). It was password-protected and only accessible by one Philips team member (DL). Before starting, panelists received reading material describing the predictive analytic tool and its performance characteristics.

 Through several meetings, six members of the Philips team with experience in critical care practice or expertise related to the predictive analytic tool identified the initial 19 outcomes to be evaluated by the expert panel. The items were developed from a review of hemodynamic literature and the expertise of the Philips team. Throughout four survey “rounds,” panelists evaluated each outcome on a 5-point scale where “5” was very relevant and “1” was not at all relevant to clinical practice. The outcomes were grouped into three categories: clinical outcomes, workflow outcomes, or population outcomes (Supplementary Materials).

**Figure 1:**
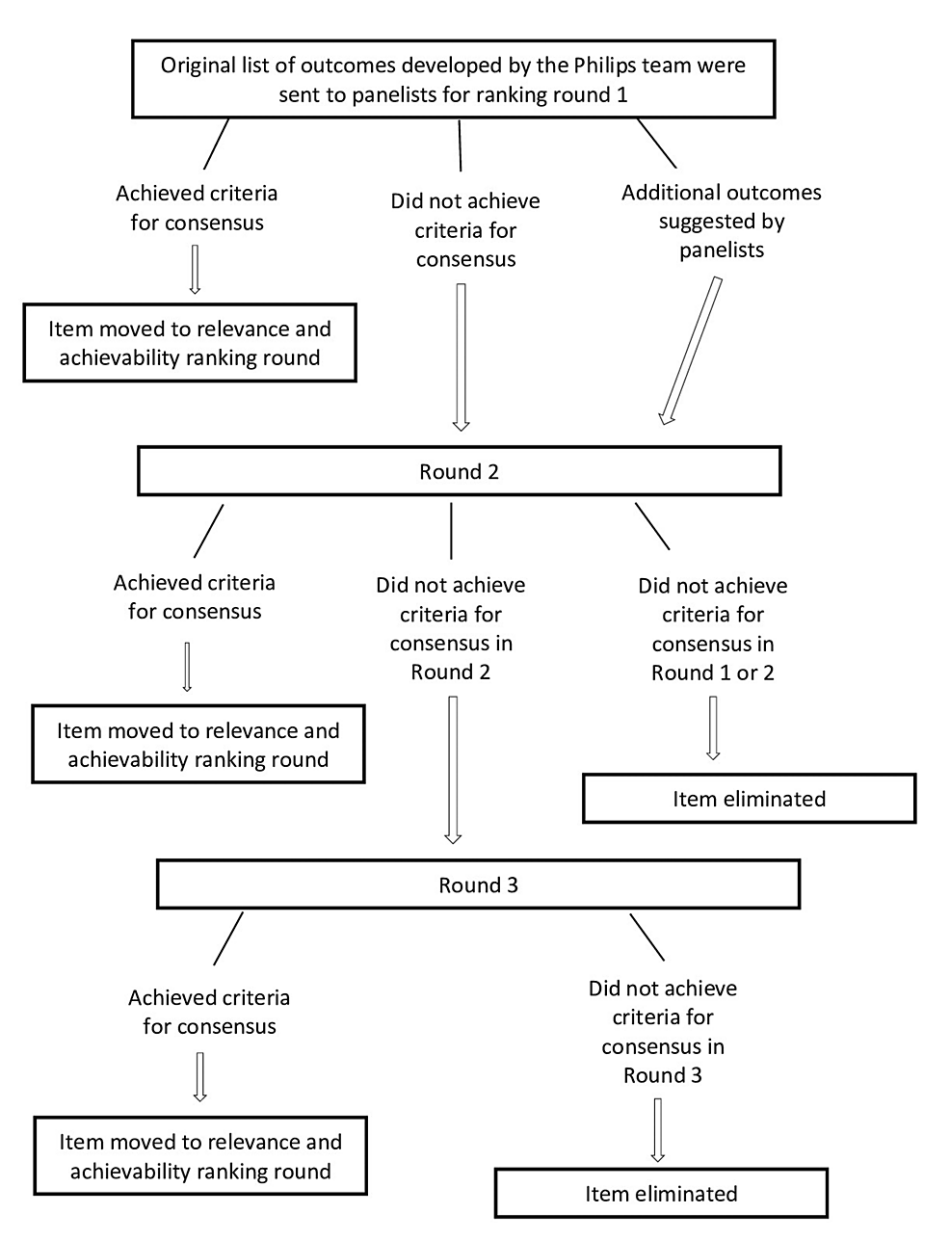
Delphi Process Flow Chart

Figure 1 shows the Delphi study flow chart. During Round 1, panelists were presented with the initial list of outcomes and asked to suggest additional outcomes they would find relevant. Thereafter, panelists ranked the 20 outcomes and provided comments for their responses. Outcomes that did not achieve consensus and new outcomes suggested by panelists during Round 1 were evaluated in Round 2. An outcome reached consensus if at least 65% of panelists ranked an outcome as either a “4 - Somewhat Relevant” or a “5 - Very Relevant.” During Round 2, panelists were presented with their prior ranking, the median ranking, and comments from other panelists during Round 1. They were then asked to re-rank each outcome on the same 5-point scale. If an outcome did not achieve consensus in Round 1 or 2, it was not re-evaluated and classified as not achieving consensus for relevance. During Round 3, panelists were presented with outcomes that were evaluated once and did not achieve consensus. These were the new outcomes suggested by the participants before Round 1 and included in Round 2. Panelists could also see their ranking from Round 2, the median ranking of all panelists, and any comments from the panelists during Round 2. Outcomes that achieved consensus during Round 3 were added to those from Round 1 and Round 2 for ranking outcomes based on relevance and achievability.

In a final ranking step, panelists ranked all outcomes that achieved consensus according to their clinical relevance based on both relevance and achievability. The median and interquartile range were calculated for each ranked outcome, as well as the correlation between rated outcomes. Agreement between raters was analyzed using Fleiss’ Kappa. All outcomes were aggregated into a single preferred order using the Kemeny-Young approach [11], a Condorcet method readily available in R (R version 4.0.3) [12].

## Results

We initially evaluated 19 outcomes to be considered as relevant outcomes associated with a predictive analytic tool to detect hemodynamic deterioration (Appendix 1). Five additional outcomes suggested by the panelists in Round 1 of the Delphi process were subsequently added in Round 2 (Appendix 2). Thirteen panelists (10 physicians and three nurses) participated in the Delphi process, and all completed each round. Demographic data of the participants is presented in Table 1. Based on the definition of consensus, at least nine of the 13 panelists had to rank an outcome as either a “4” or “5” to achieve consensus. Table 2 shows outcomes that reached consensus in rounds 1, 2, or 3 and those that did not.

**Table 1:** Demographic Data of Participants (n=13)

Characteristics	n (%)
Type of Hospital	
University Medical Center	11 (84.6)
Community Teaching Hospital	0
Community Non-teaching Hospital	1 (7.7)
Government Hospital	1 (7.7)
Years in Practice	
<5 years	0
5 to <15 years	6 (46.2)
15 to <25 years	2 (15.4)
>25 years	5 (38.5)
Board Certifications (some panelists reported more than one certification)	
Critical Care	10 (76.9)
Anesthesiology	4 (30.8)
Acute/Critical Care Nursing	3 (23.1)
Proportion of Time Spent in Direct Patient Care	
0-25%	5 (38.5)
26-50%	3 (23.1)
51-75%	2 (15.4)
76-100%	3 (23.1)

**Table 2:** Relevant/Very Relevant Consensus Rates

Outcome	Round 1	Round 2	Round 3
Achieved Consensus During the First Evaluation			
Can reduce the amount of time a patient spends in hemodynamic instability, defined as hypotension with evidence of end-organ dysfunction	100%		
Reducing the time to recognition of hemodynamic instability	100%		
Can reduce the duration of vasopressor and inotrope therapy	92.3%		
Can reduce the development of acute kidney injury	92.3%		
Can reduce the development of delirium	92.3%		
Can reduce the incidence of acute kidney injury	92.3%		
Can reduce the incidence of myocardial injury	92.3%		
Can reduce the ICU length of stay (including step-down)	84.6%		
Can reduce time on mechanical ventilation	84.6%		
Reducing the time to actionable nurse assessment or treatment (e.g., expression of concern about hemodynamic status, escalation to physician, titration or change of vasopressor/inotrope)	84.6%		
Reducing time to diagnosis of shock type	84.6%		
Decreasing ICU readmissions for hemodynamic instability when used as part of the ICU discharge readiness planning process	84.6%		
Can reduce the duration of hypotension		84.6%	
Discriminating those that require provider attention from those who do not	76.9%		
Will be correlated with time to lactate clearance	69.2%		
Achieved Consensus during 2nd Evaluation			
Will be associated with a greater decrease in SOFA score during the ICU stay	61.5%	76.9%	
improving nurse planning of workload distribution	61.5%	69.2%	
Can reduce ICU mortality		61.5%	84.6%
Can reduce direct medical costs		61.5%	84.6%
Did not Achieve Consensus			
Can reduce the incidence of unexpected vasopressor use	61.5%	61.5%	
Can be used as a marker of microcirculatory perfusion (e.g., capillary refill time, mottling scores, etc.)	53.8%	38.5%	
improving physician time and resource allocation planning during sign-off	38.5%	53.8%	
Can reduce the incidence of new-onset atrial fibrillation		38.5%	46.2%
Can decrease dependence on high-dose catecholamines for the management of hypotension		23.1%	30.8%

Round 1

Fourteen outcomes presented in Round 1 achieved consensus. Two outcomes were rated as very relevant or somewhat relevant by all panelists (“can reduce the amount of time a patient spends in hemodynamic instability, defined as hypotension with evidence of end-organ dysfunction” and “reducing the time to recognition of hemodynamic instability”). Five outcomes did not reach consensus and were re-evaluated in Round 2.

Round 2

Three additional outcomes achieved consensus during Round 2. “Will be associated with a greater decrease in SOFA score during the ICU stay” was rated as a “4” or “5” by 61.5% of panelists in Round 1 and 76.9% in Round 2. “Improving nurse planning of workload distribution” was rated as a “4” or “5” by 61.5% of panelists in Round 1 and 69.2% of panelists in Round 2. One new outcome suggested by panelists in Round 1, “can reduce the duration of hypotension,” also achieved consensus. Three outcomes did not achieve consensus in either Round 1 or Round 2 and were defined as not reaching consensus. Therefore, four outcomes remained for evaluation in Round 3.

Round 3

Two additional outcomes achieved consensus during Round 3. “Can reduce ICU mortality” was rated as a “4” or “5” and “can reduce direct medical costs” was rated as a “4” or “5” by 84.6% of panelists. The remaining two items were defined as not achieving consensus.

Prioritization/ranking round

**Figure 2:**
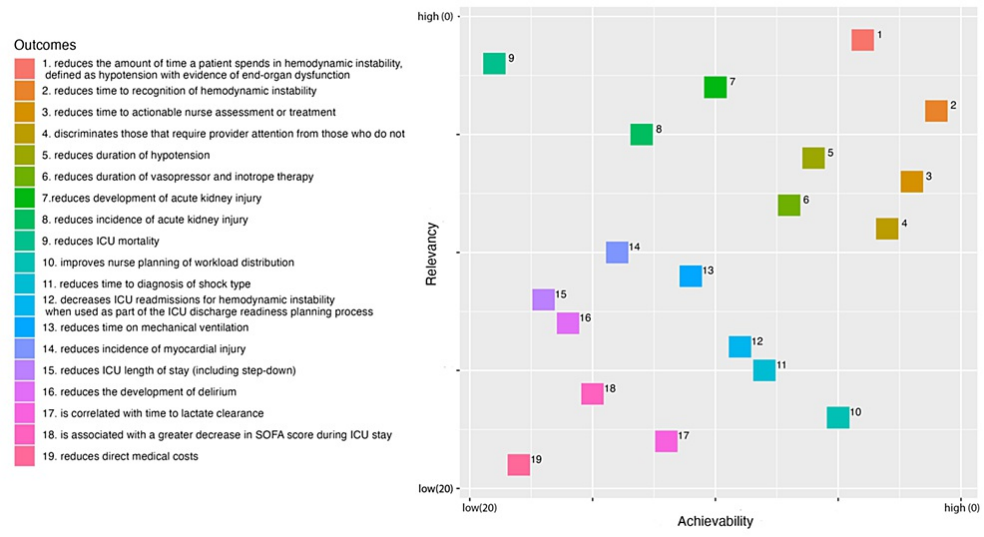
Selection Matrix for Outcome Relevance and Achievability

The most/least relevant and achievable items are listed below with Kemeny-Young Rank in parentheses. The most relevant outcomes to clinicians were “reduces the amount of time a patient spends in hemodynamic instability” (1), “reduces ICU mortality” (2), and “reduces development of acute kidney injury” (3). The outcomes achieving consensus for relevance that were ranked as most achievable in clinical studies using the HSI tool were “reduces time to recognition of hemodynamic instability” (1), “reduces time to actionable nurse assessment of treatment” (2), and “discriminates those that require provider attention from those who do not” (3). The outcomes achieving consensus for relevance that were ranked lowest on the ability to achieve in clinical studies were “reduces ICU mortality” (19), “reduces direct medical costs” (18), and “reduces ICU length of stay” (17). Figure 2 shows the selection matrix considering the rankings for relevancy and achievability of each outcome.

Fleiss Kappa was 0.0221 for the relevance ranking (p<0.001) and 0.0271 for the achievability ranking (p<0.001), indicating that there was only slight agreement on the rankings.

## Discussion

The results of this study show that the critical care physicians and nurses, as part of this expert panel, find many different outcomes clinically relevant that may be associated with a predictive analytic tool. Sixteen of the original 19 outcomes achieved consensus for relevance, and three of an additional five outcomes suggested by panelists also achieved consensus for relevance. Of the 19 total outcomes that achieved consensus, panelists believed that the HSI could improve nursing workload distribution and decrease all the following: the time of hemodynamic instability (hypotension and end-organ hypoperfusion), the time to recognition of hemodynamic instability, the time until nursing assessment or treatment, the time until the cause of shock is diagnosed, and ICU readmission. Two items were rated as relevant by all panelists during the first round. These items were “can reduce the amount of time a patient spends in hemodynamic instability” and “reducing the time to recognition of hemodynamic instability.” These results may have been expected given that the outcomes selected for evaluation have important impacts on patient care, outcomes, or workflow.

Outcomes related to workflow were also deemed relevant by panelists. Panelists felt it was relevant that the tool could discriminate those who require attention from those who do not, reduce the time to actionable nurse assessment, and improve nursing workload distribution. However, panelists did not find the outcome of improving physician time and planning during sign-off to be relevant. One panelist commented that physicians were already adept at “boiling down” patients to key summary points. Nevertheless, this tool may help accurately classify, identify, and prioritize at-risk patients. Several panelists did comment that the tool could be utilized to help focus attention on patients at the highest risk of deterioration, which could positively impact nurse staffing decisions or workflow. Two items related to vasopressor use did not achieve consensus for relevance. These items were “can reduce the incidence of unexpected vasopressor use” and “can decrease dependence on high-dose catecholamines for management of hypotension.”

Mortality was ranked as the most relevant outcome but also the least achievable outcome with this predictive analytic tool. The ability of the tool to reduce the incidence of other clinical outcomes, such as acute kidney injury, myocardial injury, and delirium, were rated as being more relevant than they were achievable. Considering the relevance and achievability of rankings together can help guide an approach to picking outcomes to study with these tools. We have presented a selection matrix (Figure 2) that provides a visual representation of each item’s ranking. The top items in this decision matrix make it clear that clinicians value outcomes related to reducing the time spent in hemodynamic instability or with hypotension and reducing the time to recognition and treatment of these events. The availability of HSI as a continuous score at the bedside would make it possible to study clinician response to HSI scores and outcomes associated with the use of the score.

If deployed properly, AI tools in critical care may enhance efficacy, productivity, workflow, and work pace [13]. A conceptual role for applying AI tools in clinical practice is their ability to enable timely detection or prediction of disease to aid in earlier patient management than is currently possible [8]. Despite the rapid growth of studies reporting on model development in the ICU, readiness to implement these models is low [14]. The Society of Critical Care Medicine’s Future of Critical Care Taskforce suggests that overcoming the barrier of evolving technology may require critical care practitioners to take a more integral role in testing, validating, and implementing smart technology and devices to improve patient outcomes and experiences [15]. However, a recent systematic review of 494 AI studies using data obtained during an ICU stay found none that reported on patient outcomes following AI model integration [14]. Implementing AI tools has been described as an “afterthought” compared with investment in model development [16]. It could be speculated that these tools are not being studied and developed in a manner relevant to bedside clinicians, which could be a barrier to the clinical adoption of a particular tool. For predictive analytic tools to be helpful to clinicians, it is vital to determine which outcomes are most relevant and achievable with predictive alerts. The ability of this tool to alert providers to impending hemodynamic instability is meaningful to clinicians and may allow for timely recognition and early intervention. Preventing hemodynamic instability is a vital first step in mitigating untoward patient outcomes. Outcomes that have been associated with hypotension development in the ICU, including mortality, acute kidney injury, and myocardial injury [17], were all determined to be clinically relevant. The HSI can alert providers one hour before a hemodynamic intervention is needed [6] and may prevent hemodynamic instability or minimize the duration.

Early awareness may allow clinicians to ensure closer monitoring, perform additional testing, or prepare interventions that may negate or minimize the period of instability. The results of this Delphi study demonstrated that clinicians find the potential impact on nursing workflow to be more relevant than the impact on physician workflow. Nurses are frontline providers consistently at the bedside and are often the first healthcare providers to recognize instability and decide on appropriate actions. Predictive analytic tools could aid in the early identification of a change in a patient’s condition, allowing for a more focused assessment [18]. Reducing the time to actionable nurse assessment or treatment is an important first step in hemodynamic monitoring and measurement. This could allow the nurse to prioritize patients for closer monitoring, prepare an intervention, or notify a provider for additional testing or intervention. The panelists found relevance in the tool improving nurse planning of workload distribution. The HSI could potentially serve as a marker of patient illness severity, identifying patients more likely to require greater care intensity and take up more nursing time. The score could assist in making nursing care assignments and identifying where greater resource intensity should be focused.

While we achieved consensus on ranking and achievability, the ordinal nature of the ranking system means that there is some variability in the relative importance of outcomes. This may be viewed as a challenge to the adoption of the HSI. However, this likely reflects regional, institutional, and individual differences in priorities and variability in what is locally achievable. For instance, hospitals with very low ICU bed availability may place a higher premium on ICU readmission rates. Other hospital systems with a high nursing burnout rate may want to prioritize HSI to make the nursing workload more equitable. Individual healthcare providers may think that HSI and other automated tools that assess hemodynamic instability should focus on decreasing the time that patients are hemodynamically unstable. In contrast, others may want a tool that improves triaging capabilities.

This study has several limitations worth mentioning. While many items were identified as relevant to clinicians, we did not assess which outcomes are most likely to lead to institutional adoption of predictive analytic tools, nor which outcomes are most relevant to patients and families. Adopting a tool may depend on factors other than the outcomes achieved. We recruited a panel of experts who practice primarily in academic health systems in the United States. Therefore, it is unclear how these outcomes may be relevant to providers from non-academic centers or other countries. The survey may not have incorporated all items relevant to clinicians; however, clinicians could suggest additional items to be evaluated during the first round. A small number of nurses were represented on the panel, and the physician panelist rankings could dilute rankings from nurses. The expert panel was too small to determine if there was a correlation between the characteristics of the panelists and rating or prioritization results; however, the panel size was in accordance with general recommendations for Delphi panel size [9]. Finally, we do not provide guidance on how to study these predictive analytic tools to achieve the identified outcomes, although prior guidance has been described [16].

## Conclusions

In conclusion, we have identified clinical and workflow outcomes, including those most achievable and relevant to clinicians that may be associated with a tool to predict hemodynamic instability in critically ill patients. The results of this study may guide anyone designing studies to evaluate outcomes associated with the use of predictive analytic tools to reduce hemodynamic instability in critically ill patients. The Delphi approach may help identify clinically relevant target outcomes. Future studies should test predictive analytical tools in the ICU that target the most clinically relevant and achievable outcomes, such as time spent hemodynamically unstable or time until actionable nursing assessment or treatment.

## Appendices

**Table 3:** Initial Delphi Survey Outcomes Scale: 5 = very relevant, 4 = somewhat relevant, 3 = neutral, 2 = somewhat not relevant, 1 = not at all relevant

In combination with a protocolized approach, this predictive analytic tool provides the clinician with insight into a patient’s likelihood of becoming hemodynamically unstable and….	
Item	Score
can reduce the amount of time a patient spends in hemodynamic instability defined as hypotension with evidence of end-organ dysfunction	
Comments:	
will be associated with a greater decrease in SOFA score during the ICU stay	
Comments:	
can reduce the duration of vasopressor and inotrope therapy	
Comments:	
can reduce the development of acute kidney injury	
Comments:	
can reduce the ICU length of stay (including step-down)	
Comments:	
can be used as a marker of microcirculatory perfusion (e.g., capillary refill time, mottling scores, etc.)	
Comments:	
can reduce time on mechanical ventilation	
Comments:	
will be correlated with time to lactate clearance	
Comments:	
can reduce the development of delirium	
Comments:	
In combination with a protocolized approach, this predictive analytic tool provides the clinician with physiologic insights into a patient’s likelihood of hemodynamic instability and can impact workflow by…	
Item	Score
reducing the time to actionable nurse assessment or treatment (e.g., expression of concern about hemodynamic status, escalation to physician, titration or change of vasopressor/inotrope)	
Comments:	
reducing the time to recognition of hemodynamic instability	
Comments:	
reducing time to diagnosis of shock type	
Comments:	
decreasing ICU readmissions for hemodynamic instability when used as part of the ICU discharge readiness planning process	
Comments:	
discriminating those that require provider attention from those who do not	
Comments:	
improving physician time and resource allocation planning during sign-off	
Comments:	
improving nurse planning of workload distribution	
Comments:	
In combination with a protocolized approach, this predictive analytic tool provides the clinician with insight into a patient’s likelihood of becoming hemodynamically unstable and….	
Item	Score
can reduce the incidence of unexpected vasopressor use	
Comments:	
can reduce the incidence of acute kidney injury	
Comments:	
can reduce the incidence of myocardial injury	
Comments:	
